# Morphea (Localized Scleroderma) Presenting With Recurrent Skin and Soft Tissue Infections: A Diagnostic Dilemma

**DOI:** 10.7759/cureus.8067

**Published:** 2020-05-12

**Authors:** Akshay M Khatri, Michael McLemore, Prashant Malhotra

**Affiliations:** 1 Infectious Diseases, Northwell Health, Manhasset, USA; 2 Dermatology, Pathology and Laboratory Medicine, Northwell Health, Manhasset, USA

**Keywords:** morphea, localized scleroderma, infection, methicillin-sensitive staphylococcus aureus, mssa

## Abstract

Morphea or localized scleroderma is reported to be triggered through diverse stimuli. We present a case of morphea that presented as a non-healing wound with superimposed methicillin-sensitive *Staphylococcus aureus* (MSSA) infection. In our case, morphea was thought to have been potentially triggered by a post-surgical infection. We discuss the potential infectious triggers and common infections that may confound the diagnosis.

## Introduction

Morphea (localized scleroderma) is a rare chronic connective tissue disease of unknown etiology. It belongs to a broader group of disorders that constitutes scleroderma, other diseases include diffuse cutaneous systemic sclerosis (DcSSc) and limited cutaneous systemic sclerosis (LcSSc). The common pathognomonic feature of these disorders is the presence of skin fibrosis and thickening [1].

The exact cause of scleroderma is not known; it is postulated to occur after exposure to an exogenous stimulus in genetically predisposed individuals [1]. DcSSc and LcSSc have been reported in conjunction with multiple environmental, viral and pharmacologic triggers [1]. Similarly, diverse factors - local trauma, radiation therapy, viral infections, Bacillus Calmette-Guerin (BCG) vaccination and silicone breast implants - have been reported as triggers for morphea [2].

We present a case of morphea that initially manifested as recurrent skin and soft-tissue infections (SSTIs).

## Case presentation

A 53-year-old Caucasian lady presented to our emergency department (ED) in October 2019 with complaints of diffuse abdominal pain worsened with movement, nausea, chills, non-healing abdominal wound and brown-colored drainage from an abdominal drain site.

**Figure 1:**
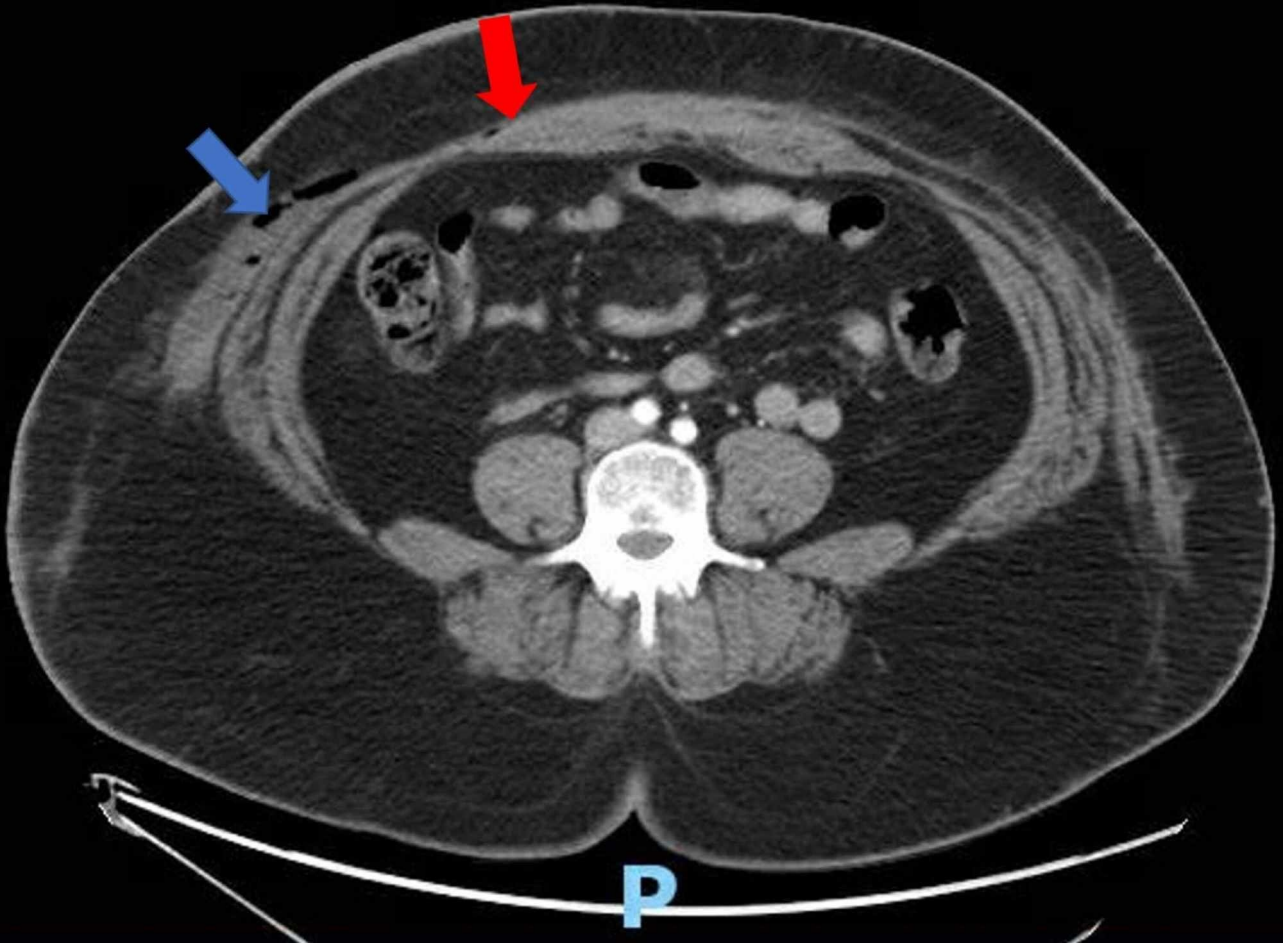
Computed tomography (CT) scan: transverse view of the abdominal cavity. Mixed attenuated fluid collection noted in the right anterolateral abdominal wall (blue arrow), with connection to the midline fluid collection (red arrow). The letter P denotes the posterior orientation of the patient.

**Figure 2:**
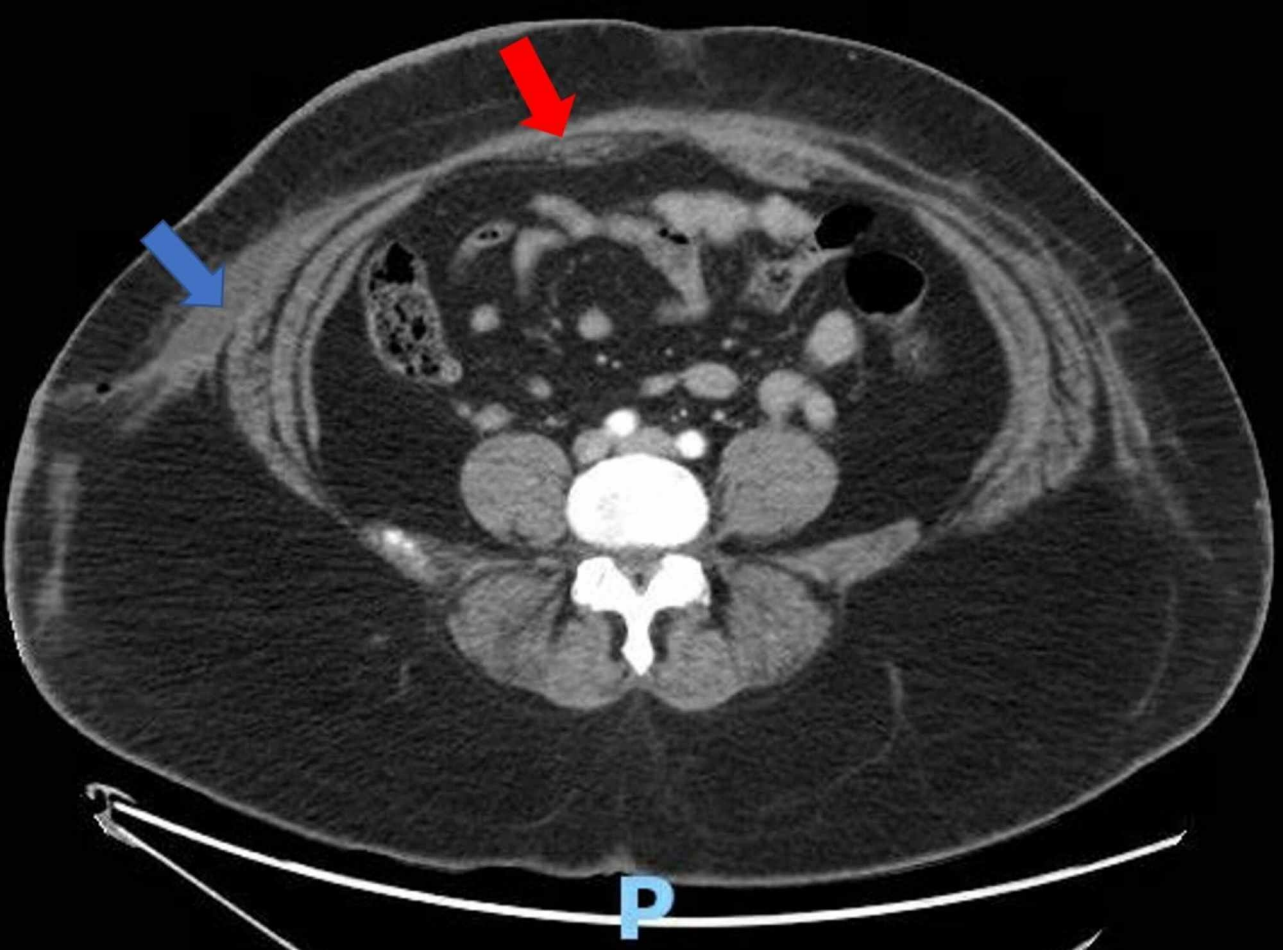
CT scan: transverse view of the abdominal cavity. Again, the mixed attenuated fluid collection in the right anterolateral abdominal wall (blue arrow) is seen. The midline fluid collection (red arrow) is better visualized. The letter P denotes the posterior orientation of the patient.

Her past medical history was significant for bilateral breast reduction mammoplasty and abdominoplasty in 2017. Her postoperative course was complicated by recurrent abdominal wall fluid collections, managed with repeat surgical drainage. After the last surgery in March 2019, she developed hyperpigmentation and ulcer formation along the anterior abdominal wall, managed with local wound care. She was re-admitted to another hospital in September 2019 with worsening abdominal tenderness. Imaging revealed a 15 cm × 2.3 cm mixed attenuation fluid collection in the right anterolateral abdominal wall, connecting to a 3.2 cm × 1.4 cm midline fluid collection (Figures 1, 2). Ultrasound-guided drainage was performed, with insertion of bilateral Jackson-Pratt (JP) drains. Wound cultures grew methicillin-sensitive *Staphylococcus aureus* (MSSA) and she was discharged on oral antibiotics, with the JP drains in situ.

On follow-up with her plastic surgeon, she reported drainage of clear brown fluid at one of the sites, with minimal JP drain output. Repeat abdominal imaging demonstrated decreased but persistent collections (9.6 cm × 1.3 cm and 3.5 cm × 1.3 cm, respectively). She was directed to the ED of our hospital for further management. Review of systems was negative except for complaints of fatigue and mild exertional dyspnea. She lived with her son and denied any toxic habits.

On initial evaluation, she was afebrile and hemodynamically stable. Systemic examination revealed no remarkable findings. Skin examination revealed a 5 cm × 5 cm tender ulceration over anterior abdomen, with surrounding mild erythema and collarettes of scale, with no active discharge or bleeding. She was also noted to have two firm hyperpigmented, indurated and excoriated plaques over the upper left and right abdomen. As per the plastic surgeon, there were no retained foreign bodies. Initial laboratory testing was unremarkable, including white blood cell count 4,740/mm^3^, hemoglobin 11.9 g/dl, blood urea nitrogen 13 mg/dl and creatinine 0.73 mg/dl.

Infectious diseases service was consulted and she was initially started on empiric cefazolin. Dermatology was consulted and punch biopsies were performed from central abdomen and edge of right abdominal ulcer. Interventional radiology was consulted and she underwent placement of an 8.5-F Dawson-Mueller pigtail catheter (Cook Medical, Bloomington, IN) into the right lower abdominal quadrant, with drainage of 30cc of clear fluid.

**Figure 3:**
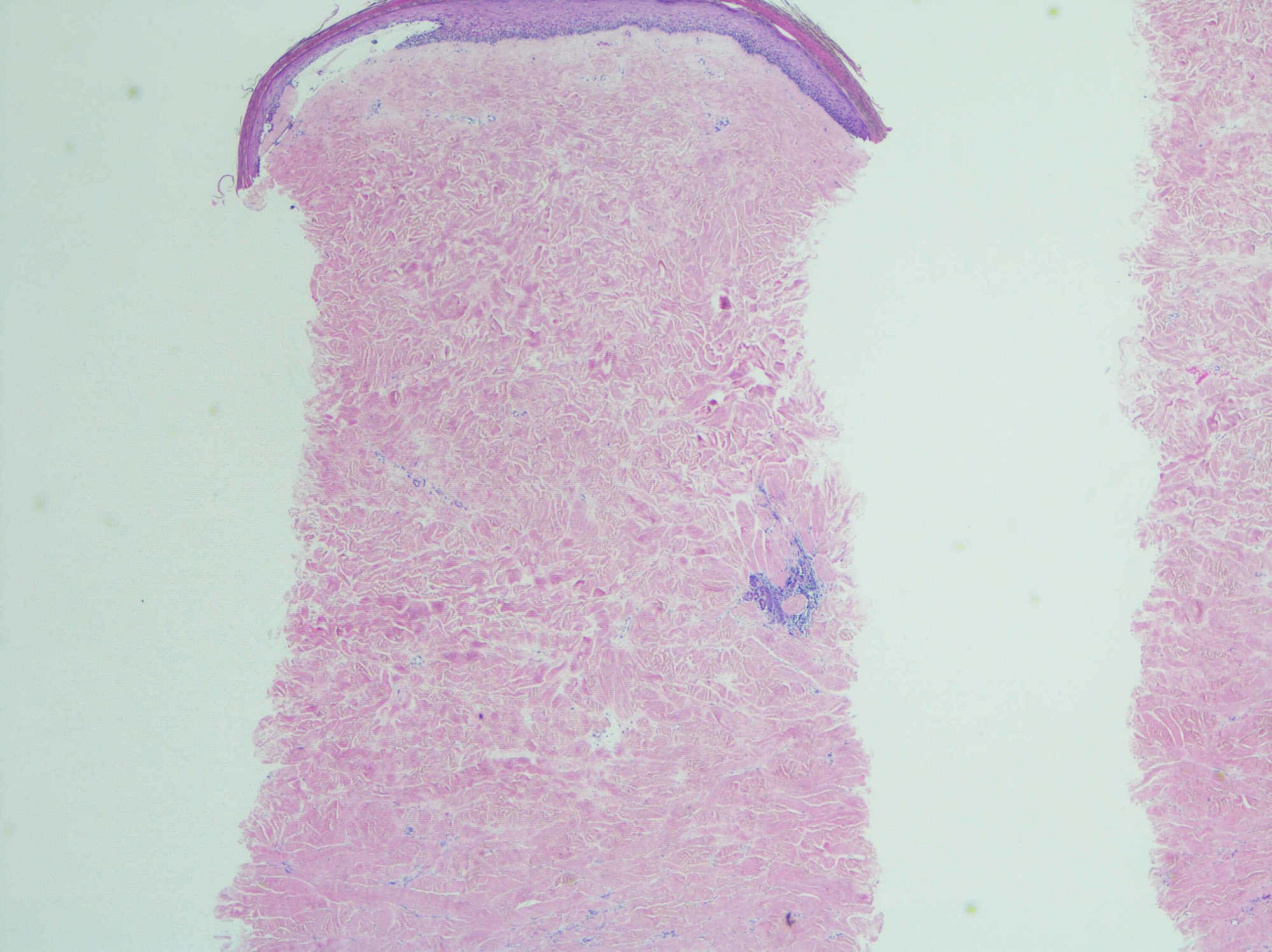
The dermis is expanded by increased, dense fibrosis (H&E, 20×).

**Figure 4:**
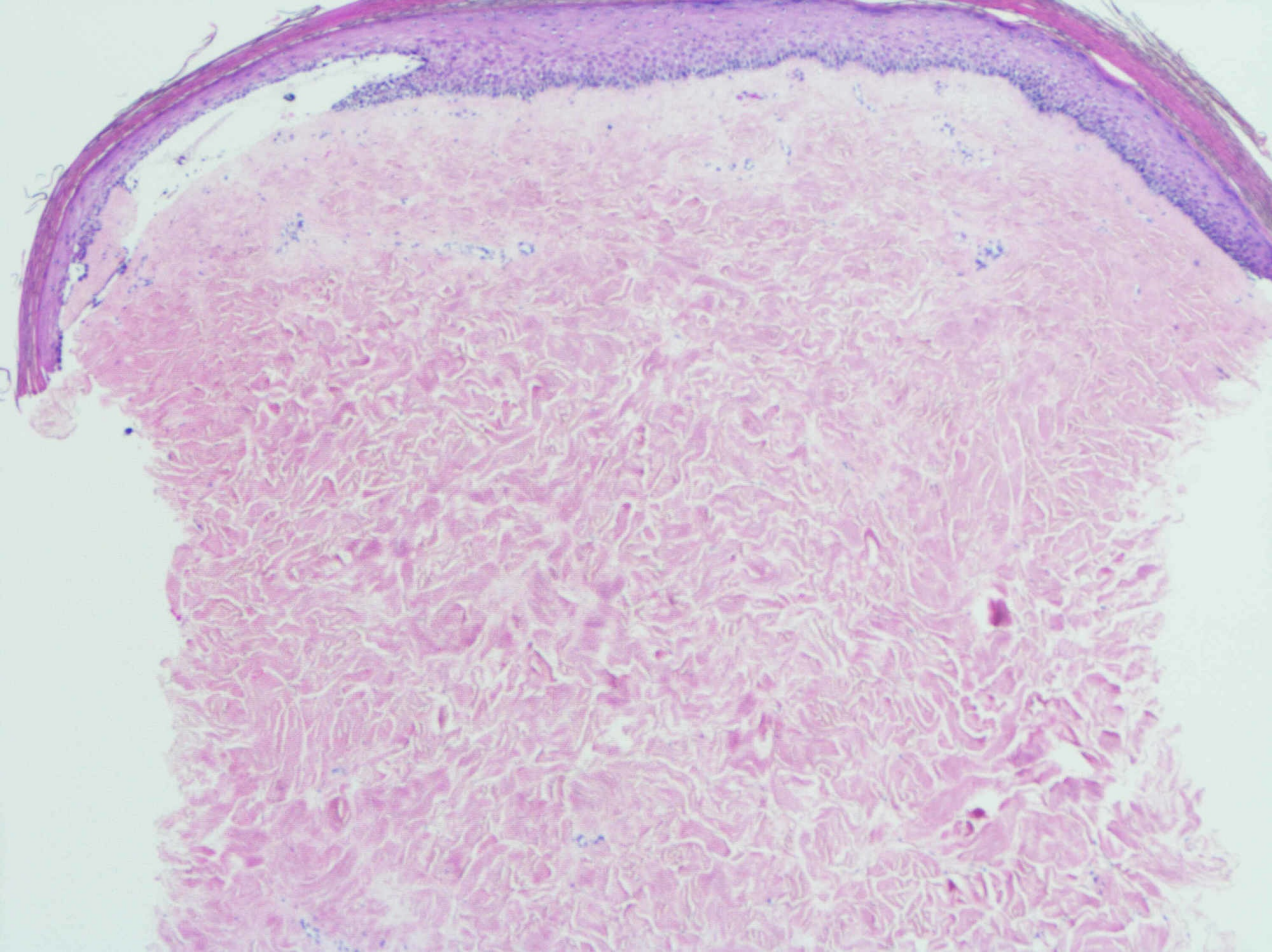
Note that the reticular collagen fibers are thickened and hyalinized. The overlying epidermis is flattened, and skin adnexal structures are diminished. Mild superficial dermal edema is noted (H&E, 40×).

**Figure 5:**
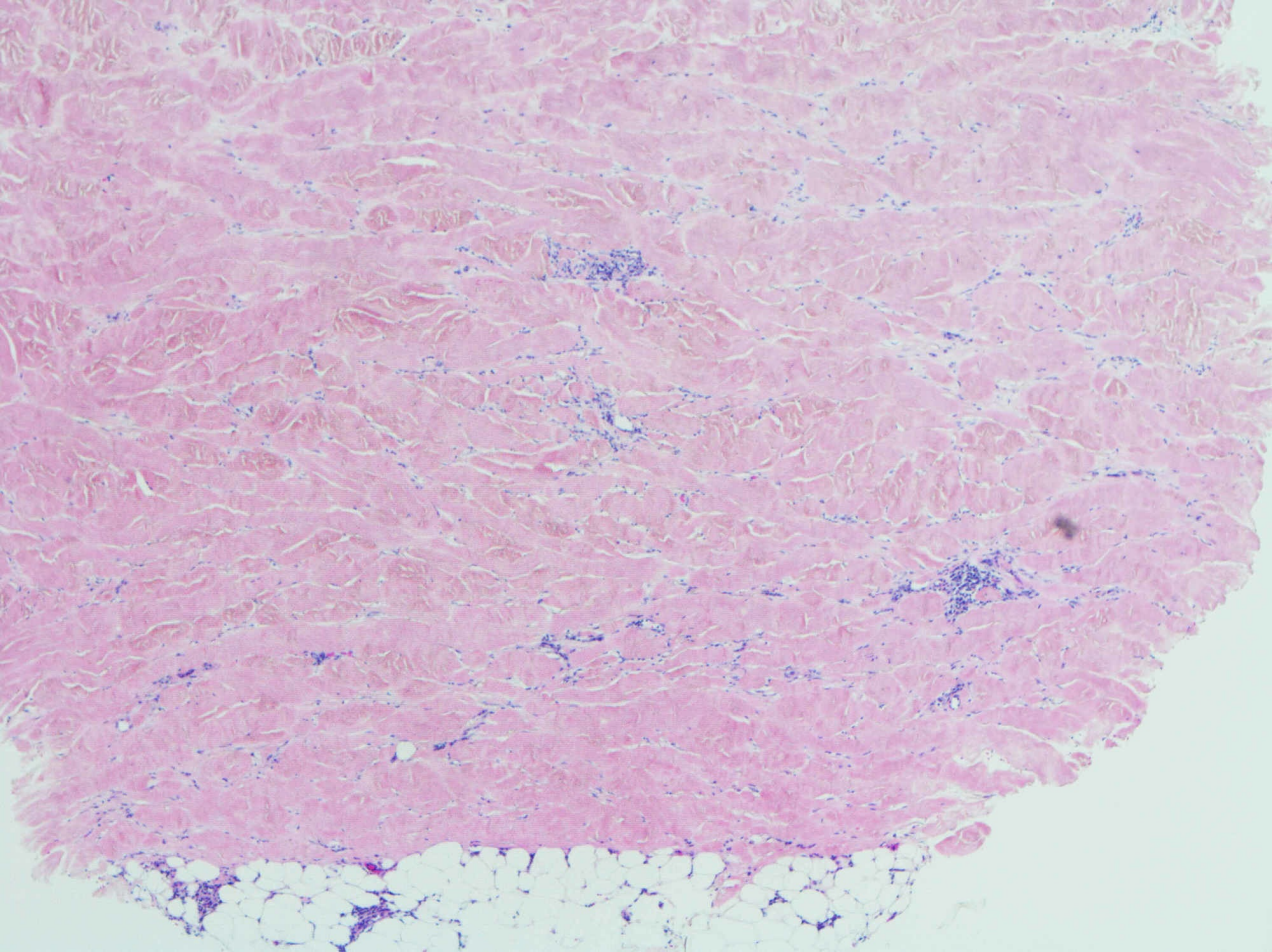
Towards the base, there is a patchy, perivascular and interstitial, chronic lymphoplasmacytic inflammatory infiltrate (H&E, 40×).

**Figure 6:**
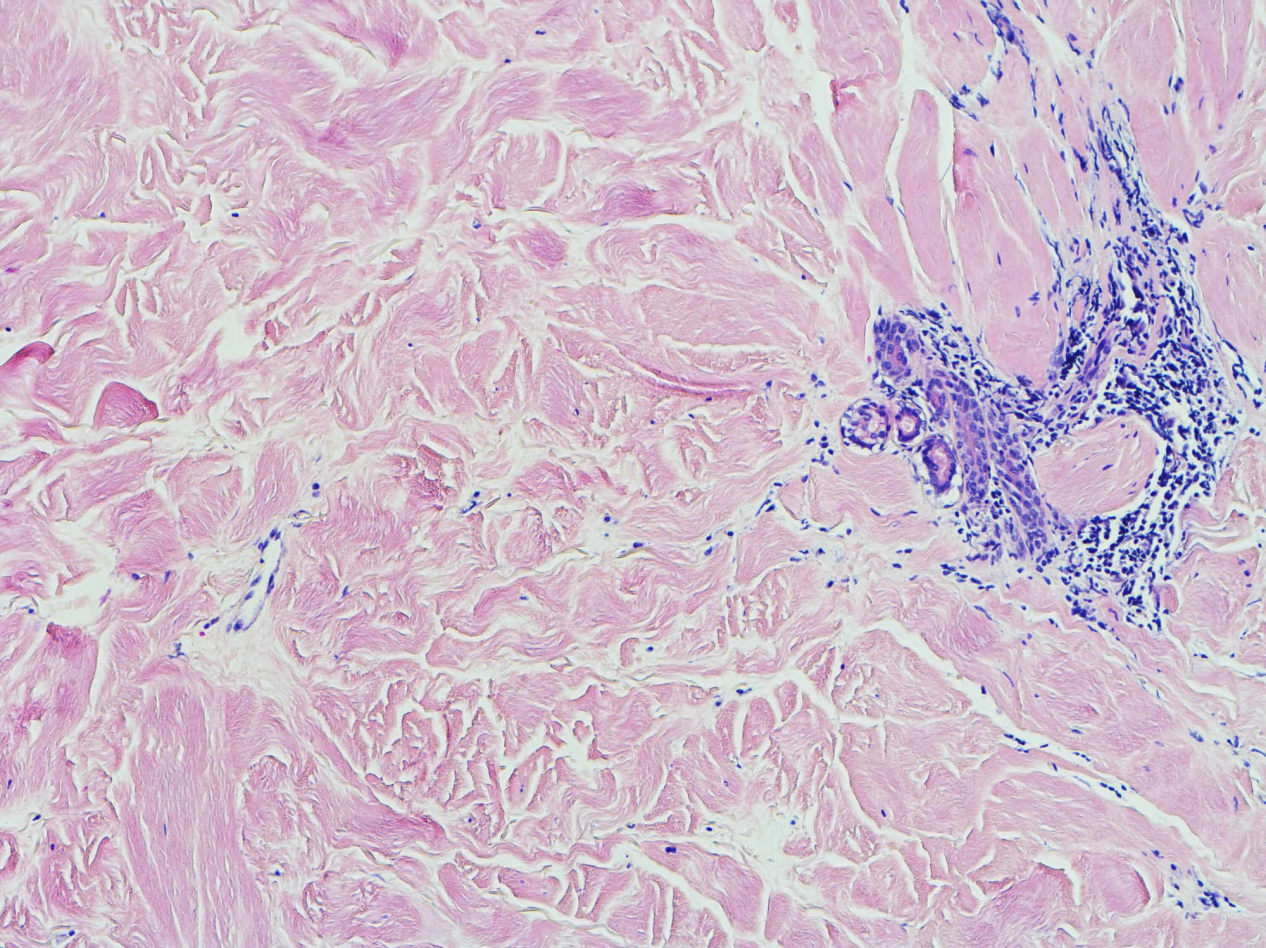
Towards the periphery, the inflammatory infiltrate is periadnexal. Note the atrophic eccrine gland (H&E, 100×)

Skin biopsy (Figures 3-6) revealed increased thickened, dense and hyalinized dermal collagen fibers, with mild, superficial and deep, perivascular and interstitial, chronic lymphoplasmacytic inflammatory infiltrate in dermis, with few eosinophils. The right abdominal wound also showed subepidermal clefts and superficial dermal edema. The dermis was expanded by fibrosis and the adnexal skin structures appeared diminished. Mild epidermal interface changes were noted. Fungal stains were negative. These changes were thought to be consistent with morphea.

**Figure 7:**
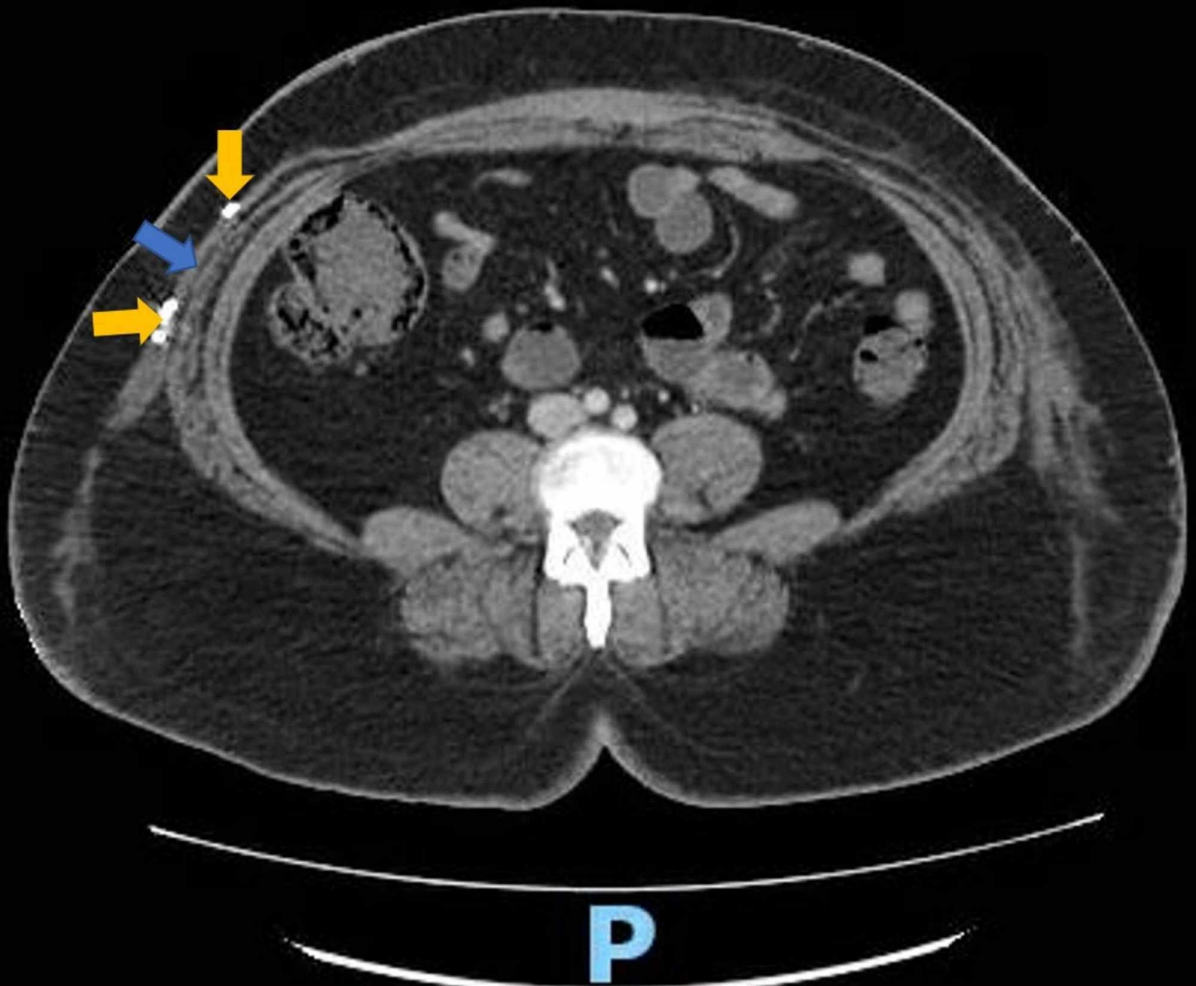
CT scan: transverse view of abdominal cavity. This CT scan was performed after placement of the pigtail catheter. The right-sided fluid collection has been drained (blue arrow) and the ends of the pigtail catheter (golden arrows) can be seen traversing the cavity. The letter P denotes posterior orientation of the patient.

Blood cultures and fluid cultures showed no growth. A repeat CT scan showed complete resolution of the fluid collection (Figure 7). She was discharged on clobetasol 0.05% ointment and oral cephalexin, with plans for outpatient dermatology, plastic surgery and infectious diseases follow-up. There was consideration for starting methotrexate, but the decision was deferred for outpatient follow-up.

## Discussion

Morphea is a rare disease. It is estimated to involve 0.4-2.7 per 100,000 people [3]. It is more predominant in females and Caucasians [1]. The peak incidence occurs in the fifth decade of life in adults and between 2 and 14 years of age in children [1]. There are five well-described subtypes: circumscribed/plaque (superficial and deep variants), linear (trunk/limb variant and head variant), generalized, pansclerotic and mixed [4]. Active morphea lesions present with signs of inflammation (erythema, induration, pain, pruritus), while inactive lesions show sclerosis and atrophy of skin [5].

Morphea may be mistaken for infections, particularly in the active stage of disease. It has been mistaken for cellulitis and tinea cruris [6,7]. Zosteriform morphea, a rare type of morphea that occurs in a dermatomal distribution, has also been reported [8]. It has been associated with non-infectious osteomyelitis of underlying bone [9].

Additionally, morphea has been reported in association with multiple infections, such as tularemia, human T-cell lymphotropic virus type 1 infection, cytomegalovirus, human immunodeficiency virus and hepatitis C virus coinfection [10-13]. The causative role of infections is unclear, some authors postulate that growth factors and immune modulators are involved in a common pathway [11,14]. Multiple studies evaluated the association of *Borrelia burgdorferi *infection with morphea, with no definite consensus. The researchers used different testing techniques in patients from different geographic areas and evaluated different strains [15-17].

The diagnosis of morphea is usually clinical, while tissue biopsy helps exclude other differential diagnoses [5]. High titers of antinuclear antibody (usually speckled pattern) have been reported in 18%-68% of patients, but autoantibody testing should be performed only if an additional autoimmune condition is suspected [5,15].

Review of the available literature revealed one recent report of morphea being mistaken for cellulitis after breast reconstruction surgery [6]. The patient presented with non-tender, non-inflamed erythematous skin lesions. She reported prior episodes of erythema over different body sites, so morphea was diagnosed clinically. However, in our case, our patient had no prior history of morphea. She had recurrent post-surgical collections, with persistent infections and non-healing wounds. Given the unclear clinical picture and lack of adequate response to antibiotics, tissue biopsy was pursued to aid in the diagnosis. We believe that a post-surgical infection with MSSA played a role in the pathogenesis of morphea. To the best of our knowledge, this appears to be the first reported instance of morphea presenting with recurrent SSTIs.

## Conclusions

Morphea may be mistaken for an infection in the acute stage. Clinicians should remember to include it in the differential diagnoses of chronic wounds with atypical, prolonged or recurrent presentations. In such diagnostically challenging cases, a multidisciplinary approach may be warranted.

## References

[1] Ferreli C, Gasparini G, Parodi A, Cozzani E, Rongioletti F, Atzori L (2017). Cutaneous manifestations of sclerdoerma and scleroderma-like disorders: a comprehensive review. Clin Rev Allergy Immunol.

[2] Shetty G, Lewis F, Thrush S (2007). Morphea of the breast: case reports and review of literature. Breast J.

[3] Kreuter A (2012). Localized scleroderma. Dermatol Ther.

[4] Laxer RM, Zulian F (2006). Localized scleroderma. Curr Opin Rheumatol.

[5] Florez-Pollack S, Kunzler E, Jacobe HT (2018). Morphea: current concepts. Clin Dermatol.

[6] Sethu C, Wong KY, Slade-Sharman D (2019). Morphea masquerading as cellulitis. BMJ Case Rep.

[7] Lee JI, Jung HY, Lee YB, Cho BK, Park HJ (2013). A case of localized scleroderma mimicking tinea cruris. Cutis.

[8] Ataş H, Gönül M, Koçak M, Gökçe A (2016). Zosteriform morphea without history of herpes zoster infection. Arch Rheumatol.

[9] Muroi E, Ogawa F, Yamaoka T, Sueyoshi F, Sato S (2010). Case of localized scleroderma associated with osteomyelitis. J Dermatol.

[10] Balestra A, Bytyci H, Guillod C, Braghetti A, Elzi L (2018). A case of ulceroglandular tularemia presenting with lymphadenopathy and an ulcer on a linear morphoea lesion surrounded by erysipelas. Int Med Case Rep J.

[11] Oiso N, Fukai K, Hosomi N, Ishii M (2003). Guttate morphoea in human T-cell lymphoma/lymphotrophic virus type-1 (HTLV-1) infection. Clin Exp Dermatol.

[12] Wong B, Piliouras P, Mortimore R, Zonta M, Tucker S (2015). Lower limb morphoea in a pregnant woman with known Grave’s disease and cytomegalovirus immunoglobulin M positivity. Australas J Dermatol.

[13] Mosquera JA, Ojea R, Navarro C (2010). HIV infection associated with scleroderma: report of two new cases. J Clin Pathol.

[14] Mihas AA, Abou-Assi SG, Heuman DM (2003). Cutae morphea associated with chronic hepatitis C. J Hepatol.

[15] Mertens JS, Seyger MMB, Thurlings RM, Radstake TRDJ, de Jong EMGJ (2017). Morphea and eosinophilic fasciitis: an update. Am J Clin Dermatol.

[16] Zollinger T, Mertz KD, Schmid M, Schmitt A, Pfaltz M, Kempf W (2010). Borrelia in granuloma annulare, morphea and lichen sclerosus: a PCR-based study and review of the literature. J Cutan Pathol.

[17] Goodlad JR, Davidson MM, Gordon P, Billington R, Ho-Yen DO (2002). Morphoea and Borrelia burgdorferi results from the Scottish Highlands in the context of the world literature. Mol Pathol.

